# Optimal Timing of Cesarean Section Following Two or More Prior Cesareans: An Investigation Into Maternal and Neonatal Outcomes (a Two-Center Study)

**DOI:** 10.7759/cureus.64291

**Published:** 2024-07-10

**Authors:** Anila Aravindan, Nilanjana Singh, Sumita Datta, Anupama Bondili

**Affiliations:** 1 Obstetrics and Gynaecology, Tawam Hospital, Al Ain, ARE; 2 Obstetrics and Gynecology, Kanad Hospital, Al Ain, ARE

**Keywords:** complication of cesarean section, timing of cesarean in previous two cesareans or more, scheduling cesarean, neonatal icu admission, multiple cesarean section, emergency and elective cesarean, optimum timing of cesarean

## Abstract

Objective

This study aimed to determine the optimal timing of elective cesarean sections for women with two or more prior cesarean deliveries by investigating maternal and neonatal outcomes across different gestational ages (37 weeks, 38 weeks, and 39 weeks).

Methods

A retrospective cohort study was conducted at Tawam and Kanad Hospitals in Al Ain, United Arab Emirates, including 435 women with previous cesarean deliveries. Data were collected on patient demographics, obstetric history, maternal complications, and neonatal outcomes, such as birth weight, appearance, pulse, grimace, activity, and respiration (APGAR) scores, neonatal intensive care unit (NICU) admissions, and length of NICU stay. The patients were divided into two groups: those with two prior cesareans and those with three or more. Outcomes were analyzed based on gestational age at delivery.

Results

Elective cesarean sections constituted 81.0% of the procedures, with no significant difference in the distribution of elective versus emergency cesareans across the studied gestational weeks (P = 0.073). Neonatal outcomes indicated healthy birth weights and low NICU admissions. For women with two prior cesareans, the NICU admission rates were 23.53% for deliveries at 37 weeks, 8.11% at 38 weeks, and 4.35% for deliveries beyond 39 weeks. For women with three or more prior cesareans, NICU admission rates were 18.18% for 37 weeks, 20.00% for 38 weeks, and 10.00% for 39 weeks. The average birth weight increased with gestational age, and NICU stays were longer for earlier deliveries (P = 0.0065 for stays > 5 days).

Conclusion

The findings suggest that the optimal timing for elective cesarean sections in women with two or more prior cesareans is 39 weeks of gestation. This timing is associated with the best neonatal outcomes, including lower NICU admission rates and healthy birth weights while minimizing the risks associated with earlier deliveries. Scheduling elective cesarean sections at 39 weeks will improve maternal and neonatal health benefits.

## Introduction

Cesarean section (CS) deliveries continue to rise globally, now constituting about 30% of births in high-income countries. This increase is notable not just for its frequency but also for the growing preference for elective CS without an immediate medical need for either the mother or neonate, sparking significant debate within the medical community about the optimal timing for these procedures [1]. While the onset of 'term pregnancy' is universally recognized at 37 weeks of gestation, opinions diverge on the best timing for elective CS, especially for women with two or more previous cesarean deliveries [2-3]. Recent evidence also highlights the variability in maternal and neonatal outcomes based on the timing of elective cesarean sections in women with prior cesareans, indicating that patient-specific factors must be considered [4].

Recent studies suggest that extending the gestational age up to 39 weeks can reduce neonatal respiratory issues, challenging prior norms that set 37 weeks as the threshold for fetal maturity [5]. This shift has led to a redefinition of 'term pregnancy' into categories: early term (37-38 weeks + 06 days), full term (39-40 weeks + 06 days), and late-term (41-42 weeks) [6], which underscores the nuanced understanding of fetal development and the timing of delivery. Additionally, studies have demonstrated that neonatal outcomes significantly improve with deliveries scheduled at 39 weeks compared to earlier gestational ages [7].

The decision on when to schedule a planned CS is critical. Elective surgery before 39 weeks has been associated with higher neonatal complication rates, whereas delaying the procedure risks the onset of spontaneous labor, potentially leading to emergency CS, and it’s associated with heightened risks for both mother and baby [8-9].

Guidelines by authoritative bodies such as the American College of Obstetricians and Gynecologists (ACOG) and the National Institute for Health and Care Excellence (NICE) recommend scheduling elective CS at 39 weeks to mitigate the risks of fetal respiratory morbidity [10-11]. However, for women with a significant surgical history, such as two or more prior cesareans, the stakes are higher. The likelihood of complications, both maternal and neonatal, escalates in the event of an emergency CS, suggesting a delicate balance in planning the timing of delivery [12-14].

Our study is designed to explore how gestational age at the time of elective CS influences the necessity for unplanned CS and its subsequent impact on maternal and neonatal comorbidity rates. By broadening the scope of our analysis to include gestational weeks 37 through 39 and differentiating between women with two versus three or more prior cesareans, we aim to provide a more detailed insight into the optimal timing for elective CS and its ramifications on health outcomes.

## Materials and methods

This study was conducted as a bi-institutional retrospective cohort analysis at Tawam and Kanad Hospitals, located in Al Ain, United Arab Emirates. It targeted women with a history of two or more prior CS who were scheduled for a repeat planned CS or encountered an unplanned CS during the study period. The study spanned from January 1st, 2019 to December 31st, 2019, and included a total of 435 patients in its analysis.

The primary objective was to examine the impact of gestational age, specifically within the range of 37 to 39 weeks, on the outcomes of repeat CS. This gestational age range was chosen to capture the nuances of elective CS timing and its implications for maternal and neonatal outcomes. The gestational age was assessed by completed weeks, clarifying that, for example, 37 weeks spanned from 37 weeks to 37 weeks +6 days. The selection of specific gestational age ranges was dictated by the practical challenge of scheduling a planned CS for an exact gestational week. Patients were divided into two groups based on their history of prior CS: those with two previous CS and those with three or more, to better understand the differential outcomes within these subpopulations.

Data collection involved a comprehensive review of medical records, including patient demographics [age, Body Mass Index (BMI)], the presence of gestational diabetes mellitus (GDM) or other comorbidities (diabetes mellitus, preeclampsia, hypertension, thyroid disorder, asthma, previous myomectomy) and detailed obstetric history, and specifics of the current CS (gestational age at delivery, elective or emergency nature of the procedure). Neonatal outcomes, such as appearance, pulse, grimace, activity, and respiration (APGAR) scores, birth weight, Neonatal Intensive Care Unit (NICU) admission, and length of NICU stay, were meticulously recorded. We also collected data regarding the indication for NICU admissions and categorized the length of stay (LOS) as either < five days or > five days. Additionally, data regarding maternal complications like postpartum hemorrhage (PPH) and scar dehiscence were collected.

Exclusion criteria were established to exclude cases with potential confounding factors, including placenta previa, accreta and its spectrum, significant maternal cardiac, renal, or lung diseases, major fetal anomalies, or multiple gestations. Additionally, any scheduled CS before 37 weeks of gestation was excluded from the analysis to focus on term and near-term deliveries.

The study utilized the cesarean logbook for initial data identification, followed by a detailed review of eligible patients' medical records for those who underwent unplanned CS. This included assessing the gestational age at delivery, indications for the unplanned CS, and the status of uterine contractions, cervical dilation, and membrane integrity at the time of surgery. Data analysis was performed using SPSS (IBM Corp. Released 2019. IBM SPSS Statistics for Windows, Version 26.0. Armonk, NY: IBM Corp). Chi-square was used for categorical data. Student's t-test and analysis of variance (ANOVA) were used for continuous variables. Differences were considered significant if p value < 0.05.

Ethical approval for this study was granted by the Ethical Research Committee of Abu Dhabi Health Research and Technology, ensuring adherence to the highest standards of ethical conduct and patient privacy protection throughout the research process.

## Results

The study delineated the outcomes of CS across two primary categories: elective and emergency procedures. The analysis extended to gestational ages between 37 and 39 weeks, focusing on women with two, three, or more previous CS. The data reveal that elective CS was more common, with 281 (81.0%) of the procedures being planned. Specifically, 157 (77.7%) of these were among women with two previous CS, and 124 (85.5%) were among those with three or more prior CS. Emergency CS accounted for 66 (19.0%) of the total, with a notable distribution across the two groups. Despite the apparent preference for elective surgeries, the difference in the distribution of elective versus emergency CS did not reach statistical significance (P = 0.073), suggesting that the number of prior CS does not significantly influence the type of CS performed within this gestational age range (Table 1).

**Table 1:** Elective versus emergency cesarean section in patients with two or three or more cesarean sections between 37-39 weeks.

Type of Cesarean Section	Previous two cesarean section	Previous three cesarean sections or more	Grand Total	P value
Elective	157 (77.7%)	124 (85.5%)	281 (81.0%)	0.073
Emergency	45 (22.3%)	21 (14.5%)	66 (19.0%)	0.073

Neonatal outcomes following elective lower segment cesarean section (LSCS) were also evaluated. The average birth weight of newborns varied slightly across the gestational weeks and previous cesarean sections but was generally healthy. The rate of NICU admissions and the length of the NICU stay are detailed below (Table 2).

**Table 2:** Neonatal outcomes and previous cesarean section for babies at 37-39 weeks of previous two, previous three or more cesarean sections. NICU: neonatal intensive care unit, LOS: length of stay.

Category	Birth weight in grams (average)	NICU Admission (No), count (%)	NICU Admission (Yes), count (%)	APGAR @ 5 mins (less than 7) count (%)	NICU LOS < five days count (%)	NICU LOS > five days count (%)	
37 weeks, previous two cesarean sections	3001.28	52 (76.47%)	16 (23.53%)	0 (0.00%)	16 (23.53%)	0 (0.00%)	
38 weeks, previous two cesarean sections	3122.47	102 (91.89%)	9 (8.11%)	0 (0.00%)	7 (6.31%)	0 (0.00%)	
39 weeks, previous two cesarean sections	3094.57	22 (95.65%)	1 (4.35%)	0 (0.00%)	0 (0.00%)	0 (0.00%)	
P value (previous two cesarean sections)	0.35	0.5	0.5	0.85	0.7	0.7	
37 weeks, previous three or more cesarean sections	2999.03	72 (81.82%)	16 (18.18%)	0 (0.00%)	16 (18.18%)	0 (0.00%)	
38 weeks, previous three or more cesarean sections	3150.2	60 (80.00%)	15 (20.00%)	0 (0.00%)	12 (16.00%)	0 (0.00%)	
39 weeks, previous three or more cesarean section	3200.5	45 (90.00%)	5 (10.00%)	0 (0.00%)	5 (10.00%)	0 (0.00%)	
P value (previous three or more)	0.32	0.48	0.48	0.82	0.68	0.68	

The study also assessed the impact of maternal factors, such as gestational diabetes mellitus (GDM) and the presence of comorbidities (diabetes mellitus, preeclampsia, hypertension, thyroid disorder, asthma), on gestational age at the time of delivery. The presence of GDM was associated with a slightly earlier delivery, particularly in women with three or more previous cesarean sections. Additionally, the data suggested a correlation between NICU admissions and maternal comorbidities (Table 3).

**Table 3:** Maternal factors influencing gestational age. GDM: gestational diabetes mellitus, NICU: neonatal intensive care unit, PPH: postpartum hemorrhage.

Factors	Mean Gestational Age (weeks) for previous two cesarean sections	Mean Gestational Age (weeks) for previous three or more cesarean section	P Value
GDM (Yes)	38.70 (0.60)	38.39 (0.64)	0.057
GDM (No)	38.11 (0.50)	37.86 (0.00)	0.01
NICU Admission (Yes)	38.28 (0.58)	38.26 (0.58)	0.894
NICU Admission (No)	38.69 (0.63)	38.30 (0.58)	0.0155
Comorbidity (Yes)	38.65 (0.61)	38.26 (0.60)	0.0154
Comorbidity (No)	38.63 (0.64)	38.30 (0.57)	0.0393
Maternal Morbidity (Yes)	38.56 (0.62)	38.43 (0.66)	0.434
Maternal Morbidity (No)	38.66 (0.64)	38.23 (0.53)	0.0006
Scar Dehiscence	37.86 (0.00)	37.86 (0.00)	1.00
PPH	38.36 (0.5)	37.86 (2.28)	0.042

NICU admission rates varied notably between elective and emergency cesarean sections, underscoring the potential neonatal risks associated with unplanned procedures. Emergency cesarean sections, regardless of the number of previous cesareans, were linked to higher NICU admission rates, highlighting the benefits of elective cesarean delivery in minimizing neonatal complications (Table 4).

**Table 4:** NICU admission versus Group 1 and 2 versus elective and emergency cesarean section. CS: cesarean section.

Category	37 weeks	38 weeks	39 weeks	Total	P value
Group 1 Elective (previous two CS)	9 (9.55%)	6 (6.38%)	0 (0.00%)	15	0.737
Group 1 Emergency (previous two CS)	7 (24.44%)	3 (10.71%)	0 (0.00%)	10	
Group 2 Elective (previous three + CS)	7 (16.13%)	6 (13.04%)	0 (0.00%)	13	
Group 2 Emergency (previous three+ CS)	1 (4.76%)	0 (0.00%)	0 (0.00%)	1	

**Figure 1:**
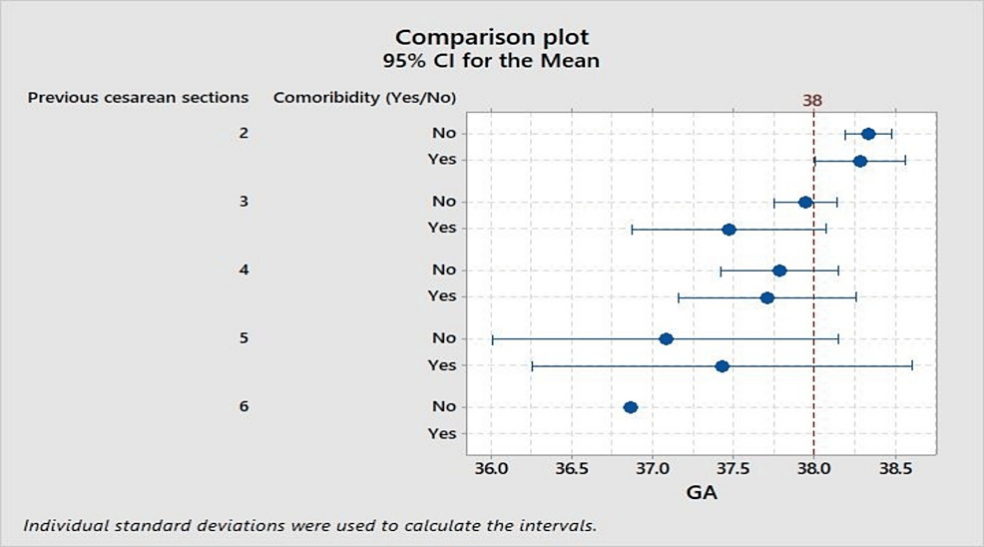
Comparison plot - 95% CI for the Mean. CI: confidence interval.

The relationship between gestational age at delivery and a range of clinical variables is illustrated in Figure 1, which displays variability in gestational age across different numbers of previous cesarean sections, with and without the presence of comorbidities. A trend towards earlier delivery is observed with more previous cesarean deliveries, particularly when comorbid conditions are present, as indicated by the confidence intervals intersecting the 38 weeks term threshold. 

**Figure 2:**
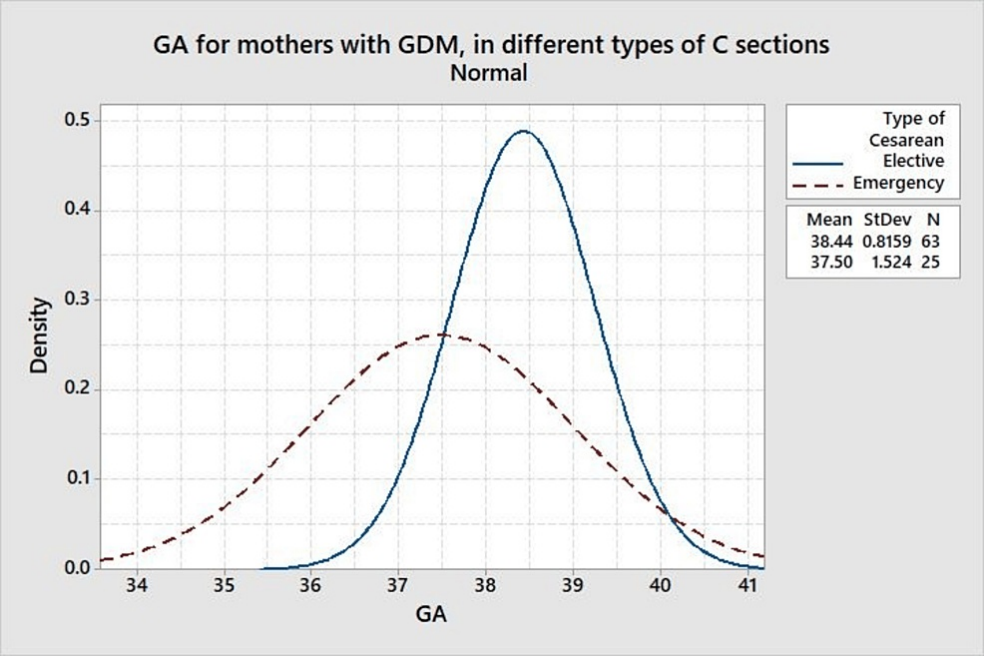
Gestational age for mothers with GDM, in different types of cesarean sections. GA: gestational age, GDM: gestational diabetes mellitus, C Sections: cesarean section.

Figure 2 a histogram compares the gestational age distribution for mothers with GDM undergoing elective versus emergency cesarean deliveries. It shows that elective cesarean sections tend to be performed closer to 39 weeks, while emergency cesarean sections occur earlier, around 37-38 weeks, highlighting a tendency to schedule elective surgeries closer to full-term for mothers with GDM. 

**Figure 3:**
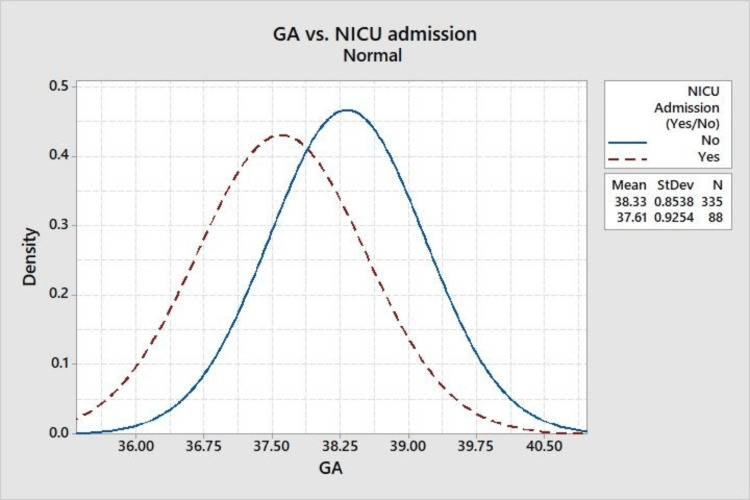
Gestational age versus NICU admissions. GA: gestational age, NICU: neonatal intensive care unit.

The correlation between gestational age and neonatal intensive care unit (NICU) admissions is presented in Figure 3 histogram. It demonstrates an earlier gestational age at delivery for babies requiring NICU care, emphasizing gestational age as a significant predictor of the need for specialized postnatal care. 

**Figure 4:**
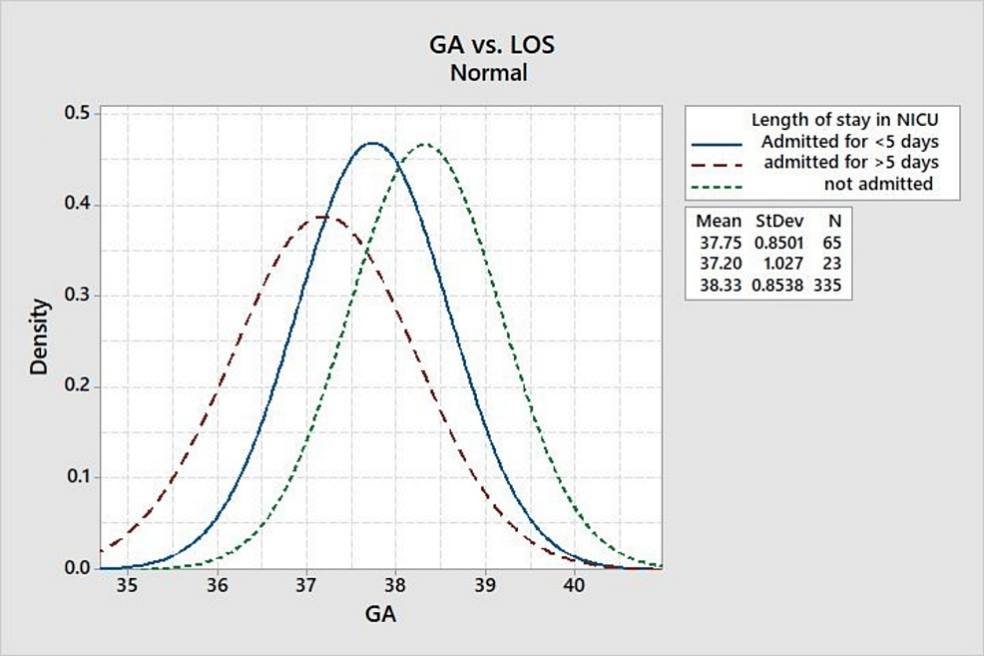
Gestational age versus length of stay in NICU. GA: gestational age, LOS: length of stay

Furthermore, Figure 4 histogram examines the length of the NICU stay, a critical indicator of neonatal morbidity. The data suggest that longer NICU stays are associated with earlier deliveries, indicating the importance of gestational timing in relation to the duration of neonatal hospitalization. 

**Figure 5:**
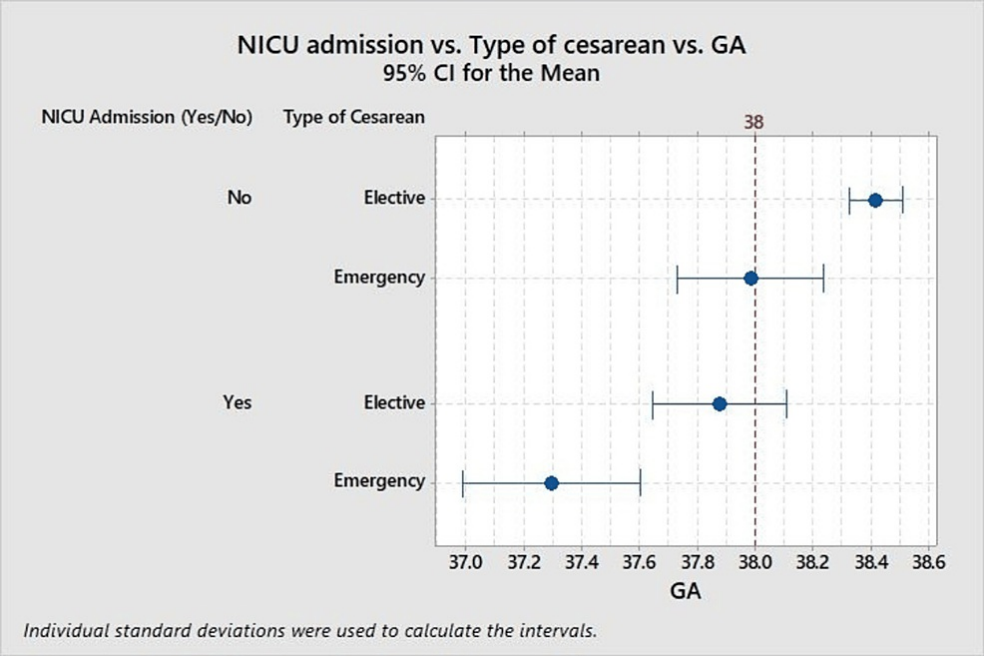
NICU admission versus type of cesarean versus gestational age. NICU: neonatal intensive care unit, GA: gestational age

Lastly, Figure 5 contrasts the type of cesarean section, elective or emergency, with NICU admission status concerning gestational age. This analysis highlights that elective cesarean sections generally correspond with later delivery dates across NICU admission statuses, suggesting more favorable outcomes in terms of gestational timing for elective procedures compared to emergency interventions. 

## Discussion

Our study comprehensively investigates the optimal timing for elective cesarean sections in women with a history of two or more prior cesareans, specifically analyzing the impact of scheduling these procedures between 37 to 39 weeks of gestation. The inclination towards elective cesarean sections, underscored by our findings, aligns with the broader discourse on reducing emergency cesarean-related complications and enhancing neonatal outcomes [1-2].

Contrary to the historical benchmark of 37 weeks as the threshold for elective cesarean delivery, our data support the extension of gestational age to 39 weeks as it was associated with decreased neonatal morbidity, aligning with contemporary research that advocates for delaying delivery to decrease neonatal respiratory complications [5]. This reevaluation of 'term pregnancy' into more granular classifications (early term, full term, and late-term) is crucial for tailoring clinical decisions to individual patient profiles [6]. Furthermore, the stratification of term pregnancies into these categories is beneficial in managing and anticipating potential neonatal respiratory issues and other complications [15].

The non-significant difference in the distribution of elective versus emergency cesarean sections across the studied gestational weeks (Table 1) suggests that the decision for elective cesarean does not substantially increase the likelihood of emergency procedures, a pivotal consideration for clinicians navigating the risks associated with unplanned surgeries [12-13]. Neonatal outcomes, particularly birth weight and APGAR scores, remained largely favorable across the elective cesarean section cohort, reinforcing the safety and efficacy of planning cesarean deliveries within this gestational window (Table 2). This finding is critical, considering the heightened risks associated with elective surgeries before 39 weeks, as highlighted by the existing literature [8-9]. Our analysis extends to the nuanced dynamics between maternal factors such as GDM and gestational age at delivery, revealing a slight predisposition towards earlier deliveries in the presence of GDM (Table 3). These insights are paramount, given the guidelines by ACOG and NICE, which advocate for scheduled cesarean sections at 39 weeks to mitigate fetal respiratory morbidity risks [10-11,16]. This is consistent with findings that highlight the complexity of managing pregnancies complicated by GDM, which often necessitates earlier intervention to mitigate risks [17].

Interestingly, the study also highlights the minimal impact of maternal comorbidities on the timing of delivery, a finding that contrasts with other research suggesting these factors could contribute to preterm delivery [18]. This discrepancy underscores the complexity of managing pregnancies with multiple prior cesareans and the necessity for a comprehensive approach that considers both maternal and neonatal factors.

The elevated NICU admission rates following emergency cesarean sections, compared to elective ones, accentuate the implications of unscheduled deliveries on neonatal health (Table 4). This further substantiates the argument for meticulously planned elective cesarean sections as a strategy to preempt the complications associated with emergency procedures.

Strengths and Limitations

The multicentre nature of our study and the way we compared the results at each gestational age make it notable. The main limitation is the low sample size and the data in our database was obtained retrospectively and entered by clinical staff. Considering some of the variables reviewed are subjective, there is a chance that data entry mistakes and discrepancies between the input and the real clinical scenario may occur.

## Conclusions

In conclusion, our study affirms the viability of scheduling elective cesarean sections at 39 weeks of gestation for women with two or more prior cesareans without any comorbidities. By doing so, it is possible to optimize neonatal outcomes while minimizing the risks associated with emergency cesarean deliveries. These findings not only contribute to the ongoing discourse on the optimal timing of cesarean sections but also underscore the importance of individualized patient care, reinforcing the need for further research to refine elective cesarean scheduling practices.
